# High turbidity levels alter coral reef fish movement in a foraging task

**DOI:** 10.1038/s41598-021-84814-5

**Published:** 2021-03-19

**Authors:** Cait Newport, Oliver Padget, Theresa Burt de Perera

**Affiliations:** Department of Zoology, University of Oxford, Zoology Research and Administration Building, Mansfield Road, Oxford, OX1 3SZ UK

**Keywords:** Behavioural ecology, Marine biology, Marine chemistry

## Abstract

Sensory systems allow animals to detect and respond to stimuli in their environment and underlie all behaviour. However, human induced pollution is increasingly interfering with the functioning of these systems. Increased suspended sediment, or turbidity, in aquatic habitats reduces the reactive distance to visual signals and may therefore alter movement behaviour. Using a foraging task in which fish (*Rhinecanthus aculeatus*) had to find six food sites in an aquarium, we tested the impact of high turbidity (40–68 NTU; 154 mg/L) on foraging efficiency using a detailed and novel analysis of individual movements. High turbidity led to a significant decrease in task efficacy as fish took longer to begin searching and find food, and they travelled further whilst searching. Trajectory analyses revealed that routes were less efficient and that fish in high turbidity conditions were more likely to cover the same ground and search at a slower speed. These results were observed despite the experimental protocol allowing for the use of alternate sensory systems (e.g. olfaction, lateral line). Given that movement underlies fundamental behaviours including foraging, mating, and predator avoidance, a reduction in movement efficiency is likely to have a significant impact on the health and population dynamics of visually-guided fish species.

## Introduction

Animals process physical and chemical stimuli using a range of sensory systems, which are finely tuned to the signals that they have evolved to detect. Increasingly, anthropogenic activities are causing ‘sensory pollution’ which can mask or alter stimuli, and lead to changes in the detection and interpretation of sensory information, damage to the sensory systems themselves, or attentional deficits^1–10^. The use of other sensory modalities may help animals compensate, however in some cases, sensory pollution impacts multiple sensory systems^4^ or the sensory information gained by an alternate system is not equal to that of the primary system. The impacts of sensory pollution on fish is a relatively new field but there is already evidence that auditory^1–3,11,12^, olfactory^5–8^, and chemical^9,13,14^ impairment can have an impact on animal behaviours such as communication^10,15^, predator avoidance^2^, stress levels^16^, and orientation^17^. Here, we aim to describe the effects of visual sensory pollution (via increased turbidity) on the movement of fish during foraging. As successful foraging underpins survival and success, it is crucial to understand factors which may reduce its efficacy.

Particulate matter in water causes visual pollution by increasing the scattering of light and reducing visibility. The altered optical properties of water can affect the distance from which visual information can be detected and also the spectral quality. Many aquatic ecosystems naturally face a high degree of variability to their visual environment which can occur over short, medium, and long-time scales. For example, tidal changes can increase turbidity over the course of hours, seasonal variations can occur over several months, and stochastic events such as storms and cyclones can be infrequent but develop and dissipate relatively rapidly. Increasingly, systematic rises in turbidity are being caused by anthropogenic perturbation^18^ in habitats where fish may not be accustomed to such a high degree of variation and therefore likely to be negatively impacted^19^. This may be particularly problematic in shallow coastal habitats, including coral reefs^20^, where vision is a significant and important sensory system that guides a wide range of behaviours^21–23^. It is estimated that > 60% of the world’s reefs are currently directly threatened by local sources including coastal development, watershed-based pollution and structural damage^18^. The potential impacts of increased sediment suspension to photosynthetic species have been considered e.g.^24–27^ but there is increasing evidence that fish are also negatively impacted.

Increasing turbidity has been shown to affect the development of visual systems and the behavioural ecology of fish from a wide range of habitats. Freshwater cichlids (*Aequidens pulcher*) exposed to variation in light during development possess altered photoreceptor composition^28^ and spectral sensitivity, resulting in changes to visual behaviour^29,30^. Guppies (*Poecilia reticulata*) have been found to be less active and more often solitary in high turbidity, potentially making them more vulnerable to predation^31,32^. For species of planktivorous reef fish, turbidity can cause increased attack rates but decreased attack success^33^. Other behavioural effects of turbidity include changes to anti-predator behaviour^34–38^ and predation rates^39–41^, reaction distance to visual stimuli^38,42–44^, response to sensory information e.g.^45^, foraging success^33,38,46–50^, and juvenile settlement and development^6,50^. However, some species can adapt or acclimate to changing visual conditions. For example, guppies raised in turbid water displayed a shift in spectral sensitivity and an increase in activity^51^, and several other fish species shift prey target species^52–54^.

Effective and efficient movement underpins a wide range of essential fish behaviours including foraging, navigation, mating, shelter use and territoriality, and predator avoidance. Visually guided movement, and more broadly navigation, is likely to be particularly sensitive to changes in visibility caused by turbidity as it will decrease the distance at which orientation cues can be detected. There is increasing research interest in the mechanisms of fish navigation and its sensory basis across unfamiliar or large spatial-scales (e.g. polarised light, sound, smell, magnetic compass). However, there has been comparatively little research on fish movement within small spatial scales. Thus far, at least eight species of freshwater^55,56^ and marine^57–61^ fish are known to use visual cues for spatial orientation, but the crucial question of how this behaviour is affected by changes in visibility conditions remains unanswered. In this study we have conducted for the first time, a detailed analysis of the movement of fish in changing turbidity conditions during a foraging task, as well as developed a novel method to measure and characterise differences in trajectories.

Picasso triggerfish (*Rhinecanthus aculeatus*) typically forage within a territory and frequently return to a small coral shelter. Time spent foraging and eventual success rate is likely reliant on their readiness to leave their shelter as well as their ability to detect food to return to their shelter. Using a task in which fish have to leave a shelter and search for food pots located at fixed positions in an open area, we tested how turbidity altered the time taken to leave the refuge, as well as the movement efficiency during foraging. Fish were tested in high (40–68 Nephelometric Turbidity Units (NTU)/ 154 mg/L) and low (0.4–1.4 NTU/ 0 mg/L) turbidity conditions. Typical turbidity levels can vary by site but low levels range from about 0.5–5 NTU, while higher levels are 10–100 times that e.g.^62,63^. While a meta-analysis found that many visual behaviours can be altered by even low turbidity levels (3–5 NTU)^64^, particularly in coral reef species e.g.^33,36,65^ other species and behaviours were altered at higher levels (> 30 NTU) e.g.^6,44,47,51,52,66,67^. The turbidity range we chose has been previously measured on the Great Barrier Reef where our study species has been found^62^.

## Materials and methods

### Subjects

All experimental protocols and husbandry were approved by a Local Ethical Review Committee of the University of Oxford’s Department of Zoology, and methods were carried out in accordance with relevant regulations and the code of practice for the care and use of animals for scientific purposes. A total of seven Picasso triggerfish (*Rhinecanthus aculeatus*) were used in this experiment, however the data from only four fish were used in the analysis as three fish showed signs of stress and did not participate in experiments. Fish were purchased from local suppliers and were wild caught. Fish were housed in individual aquaria (36 × 104 cm) in a flow through marine aquarium system and fed a mixed diet of pellets (Ocean Nutrition, Formula One Marine Pellet), fish, krill and cockles. All fish had live rock and a commercially available fish shelter in each tank. All fish were experimentally naïve prior to this experiment.

Prior to experimentation, fish were given five acclimation sessions on consecutive days in the experimental tank. Fish were transferred from their home tank and placed in the experimental tank for one hour. They were then given a break of one or two days before experiments began. Four of the fish were categorized as small (total length: Fish 31P = 93 mm, Fish 32G = 92 mm, Fish 37K = 96 mm) and four were large (total length: Fish 38I = 165 mm, Fish 39J = 155 mm, Fish 40M = 130 mm, Fish 41N = 126 mm).

### Experimental tank

Experiments were run in a separate experimental tank (Supp Fig. 1). The overall tank size was 600 × 1000 mm, and was divided into three sections with acrylic boards. Water was pumped around the tank (Eheim Compact 2000) to maintain flow and aeration. The first Sect. (600 mm × 215 mm) was considered a refuge for the fish and had two shelters that were identical to those found in home tanks. Two water inlet hoses were fed into this section. An opaque white wall with a guillotine door divided the refuge from the main testing area. This wall had two perforated sections to allow water to flow through the tank. The main testing area (600 mm × 740 mm) had an acrylic board on the bottom with hook and loop dots (Velcro Brand, 19 mm coins) which were used to hold six food pots in place (see below for food pot details). There were six possible positions for the food pots (A–F). The first three (A–C) were arranged in a row 19 cm from the entrance door. The second row (D–F) were 38 cm back from the first row. A final Sect. (600 mm × 40 mm) was partitioned with a perforated grey board and contained an outlet hose.

Food pots were small plastic petri dishes (diameter: 35 mm, depth: 10 mm) filled with agar agar jelly that held a single food pellet in the centre. Food pots were made by mixing 3 g of agar agar powder (Special Ingredients Ltd) with 225 mL of reversed osmosis filtered (RO) water and following package instructions. Approximately one teaspoon of mixture was added to each petri dish with a single pellet, and the mixture was allowed to set. Fresh pots were made daily. All fish were given opportunities to explore these pots in their home tank prior to the experiment, and all subjects were able to find and remove the food pellet from within.

The water in the experimental tank was removed at the end of every day and refilled from the main flow through system every morning to ensure that the water in the home tanks and the experimental one had the same temperature and chemistry, thereby reducing possible fish stress.

### Experimental procedure

At the beginning of every trial, a fish was caught with a net and a bucket and moved from its home tank to the holding area of the testing tank with the guillotine door down. A video camera (GoPro Hero 5. Resolution: 1080p, frames per second: 60, field of view: linear) placed above the tank was turned on and a turbidity measurement was made (Hach 2100Q portable turbidity meter). The fish was given five minutes to acclimate to the new tank after which a second turbidity sample was taken. The door was then opened (trial start time), allowing the fish to enter the main tank. The time at which the fish first entered the main tank was recorded (latency). The time and order of the food dishes the fish ate were recorded by an observer. When the final pellet was consumed, a third turbidity sample was taken (actual time). The fish were given a full 30 min, including acclimation time, in the tank and their overall time spent in the refuge and main tank areas were recorded. At the end of the 30 min, a final turbidity sample was taken.

The water for turbidity samples were taken from the middle of the water column in the middle of the tank. Vials were rinsed in RO water between uses and the exteriors were dried and polished. For each sample, three measurements were taken. The first two were identical repeated measures, but the vial was inverted once before the third measurement was read. The mean of the three measurements was used as the turbidity level for an individual sample.

Two testing conditions were run: (1) low, and (2) high turbidity. Each fish completed 10 trials for each condition. Low turbidity trials had nothing added to the water taken from the main flow through systems. High turbidity trials had 12.5 g of calcium carbonate (Intra Laboratories Ltd) added to 81L of water. This was added before fish were introduced to the experimental tank. Other materials such as Kaolin^31,34^ and Bentonite clay^32,33^ have been used in similar experiments with fish. All three materials have small particles which are easily suspended in water. Calcium carbonate was chosen for this experiment because it is naturally present in marine environments and can be safely added to marine aquarium systems. There is no evidence that high levels will cause harm or undue stress to the fish.

A single trial was run per day to standardize satiation levels, and trials were run every weekday where the treatments alternated (e.g. day 1: high, day 2: low, day 3: high, etc.). Therefore fish were only exposed to high turbidity for 30 min every second day during weekdays. Half the fish started with a low turbidity trial, while the other half started with high turbidity. Low turbidity trials were between 0.4 and 1.4 NTU and high turbidity was between 40 and 68 NTU. In the high turbidity trials, over the 30 min testing period, the levels would decrease, even with flow driven by a pump running through the tank. We found turbidity decreased by approximately 27% (sd: 5.25) throughout an entire 30 min trial (Supp. Fig. 2), but even the lowest levels measured were still 40 times higher than the low turbidity condition. The mean turbidity in high turbidity trials when the door was opened (trial start) was 50.95 NTU (sd: 3.78). The mean turbidity when all food had been eaten was 49.67 NTU (sd: 4.23). Given the relatively small difference in turbidity during this period, which is the focus of our analysis, changing turbidity was not included as a factor in data analyses.

For the smaller fish, the six possible pellets are adequate for daily feeding, but the larger fish were given supplemental pieces of fish or krill at the end of every day. On weekends, small fish were given pellets and fish pieces on one day only and not fed on the second day. The larger fish were also given pellets and fish pieces on the first day and a large cockle in the shell on the second day.

### Foraging efficiency

Each trial was recorded on video and also observed by the experimenter. For each trial, the experimenter recorded the turbidity levels, latency to consuming each pellet, the sequence of pellet consumption, the number of times the fish went in and out of the acclimation area, and whether the fish went into the shelter at all while in the acclimation area. The video covered the main testing area of the tank, and was used to confirm the experimenter’s observations. A custom written program (Matlab) was used to track and analyse the fish movement trajectories in each trial. Through a background subtraction method, the entire body of the fish was identified and the centroid of this collection of pixels was recorded. The fish was tracked at a rate of two frames per second. All videos with the points tracked were watched by the experimenter to detect and fix any errors in tracking. Even in the high turbidity conditions, any error rate associated with detecting the fish will come from differences in the outer edge of the fish. As the point measured was the centroid of the fish, potential differences in overall area detection do not have a meaningful impact to the analysis and the fish was detected in all frames. Six test parameters were identified: (1) exploration latency (time fish left the refuge), (2) total foraging time (time fish finished last pellet), (3) actual foraging time (time fish finished the last pellet—exploration latency), (4) total distance travelled (measured from when fish left refuge for the first time to when the final pellet was eaten), (5) travel efficiency (total distance travelled during foraging/actual foraging time), and (6) E_max_ which is the maximum expected displacement of a trajectory as a function of the number of steps^68,69^. Time was measured based on video analysis to the nearest half second (two frames per second). Distance measurements were calculated based on changes in calibrated pixel values. The maximum expected displacement was calculated using the E_max_ function in the R:trajr package; larger values (approaching $\infty$) indicate straighter paths^69^.

Differences between treatments for each test parameter were analysed using linear mixed effects models (‘lme4′ package in R^70^). The turbidity level (high vs low) were included in the model as fixed effects and individual fish ID was included as a random (intercept-only) effect to account for repeated measures from the same fish. Trial number was also included as a fixed effect (an ordered, quasi-numeric variable) to account for the possibility of learning over the course of the experiment.

$${\text{Test}}\;{\text{parameter}}\;\sim {\text{Turbidity}}\;{\text{level}}\; + {\text{Trial}}\;{\text{number}} + \left( {1|{\text{ Fish ID}}} \right)$$

The ‘jtools’ package in R ^71^ was used to calculate the fixed effect regression estimate and 95% confidence interval of the model (the model estimate of the difference between treatment means). A likelihood ratio test was used to test whether a model with and without turbidity treatment as a fixed effect better explained the variation in each response variable.

### Movement analysis

There are a range of methods that can be used to characterize movement trajectories (e.g. tortuosity, similarity to the beeline, sinuosity), but these generally assume that subjects have freedom of movement, or that their movement is directed towards a single goal. In our experiments, subjects were limited by the relatively small area of the tank and there were six possible reward locations causing the fish to frequently change directions. To deal with this, we analysed how rapidly new areas were explored by the fish, and as a measure of movement efficiency, we analysed how this metric increased per unit of distance travelled. To do this, a minimum convex polygon (MCP) was drawn around the fish track at each half-second time point, and the area was recorded. To disentangle the spatial and temporal components of space exploration (i.e. were differences just caused by fish in one treatment performing the same search movement faster), we divided the measured MCP by the distance travelled by the fish up to that point. This gave us a measure of area explored per unit distance travelled, and identified if a more convoluted path was taken to explore a given area between two points. See Supp. Video 1, for a visual example of how a trajectory results in MCP values. This described how movement of the fish was translated into exploration in new parts of the tank throughout the trial. These measures allow us to assess whether fish alter their exploration strategy in turbid conditions, and whether this manifests as differences in exploration efficiency.

The distances travelled by fish between each sampling time point (two frames per second) were calculated and, since the frame rate remained constant, this was a measure of relative mean speed between relocations. Linear mixed effects models were used to test for an effect of treatment on speed, with turbidity level as a predictor, trial number as a fixed effect, and fish ID as a random (intercept only) effect.

We employed linear mixed effects models to test for an effect of treatment on MCP and MCP per unit distance as response variables. Predictor variables were the interaction between treatment and time elapsed, trial number as a fixed effect, and fish ID as a random (intercept only) effect to account for repeated measures. This model was compared with a null model, containing only additive effects of treatment and time to look for an effect of treatment on the rate of increase in the response variable over the course of the experiment. So that all trials were comparable, the minimum time taken by any fish was used, therefore only the first 19 s were analysed.

A final analysis was conducted to determine whether fish encountered food more often than would be expected by chance given their movement parameters (speed and turning angle). The idea is that spatially coherent movement, aided by sensory inputs, could improve fishes’ ability to locate food pots. Whether encounters with food pots is more likely than chance should be indicative of whether fish in low and high turbidity water were able to direct movement and use senses to encounter food better than random searching. To measure this, we compared real fish trajectories with pseudo-random trajectories where distances travelled and turning angles were identical to real fish but turning direction (clockwise or counter clockwise), and the start direction (when leaving the refuge area) was randomized. Where pseudo-random fish movement would take them out of the tank area, a turning angle was randomly drawn until the resulting trajectory remained within the tank area. This allowed the correlation between step length and turning angle to be retained from the real fish. Food encounter rate was measured as the number of occasions that trajectories came within 30 mm of a food pot per time step spent away from pots (time spent feeding was not counted). This encounter rate was then compared between treatments and between real and simulated fish. Each fish trajectory was randomized, giving a pseudorandom fish for each trial in each treatment. Generalized Linear mixed effects models (with binomial error structure) were used to estimate encounter rates and their confidence intervals for pseudorandom fish from both treatments, taking account of repeated measures from individual fish.

## Results

The smaller fish all exhibited stress by never eating in a trial (32G), refusing to leave the refuge (31P), or showing other behaviours associated with stress (such as rapidly and repeatedly swimming along the wall of the aquarium) (37 K; 38I, trials 2–3). Hence the small fish were excluded from the experiment, and only the four larger fish were included in the final analysis. In a limited number of these trials (n = 3), the subject did not leave the refuge area (Fish 41N, trial 4, high turbidity) or eat all the food pellets (Fish 41N, trials 2, 4, high turbidity; Fish 38I, trial 1, high turbidity). The missing data from these trials were not included in the analyses.

### Foraging efficiency

**Figure 1:**
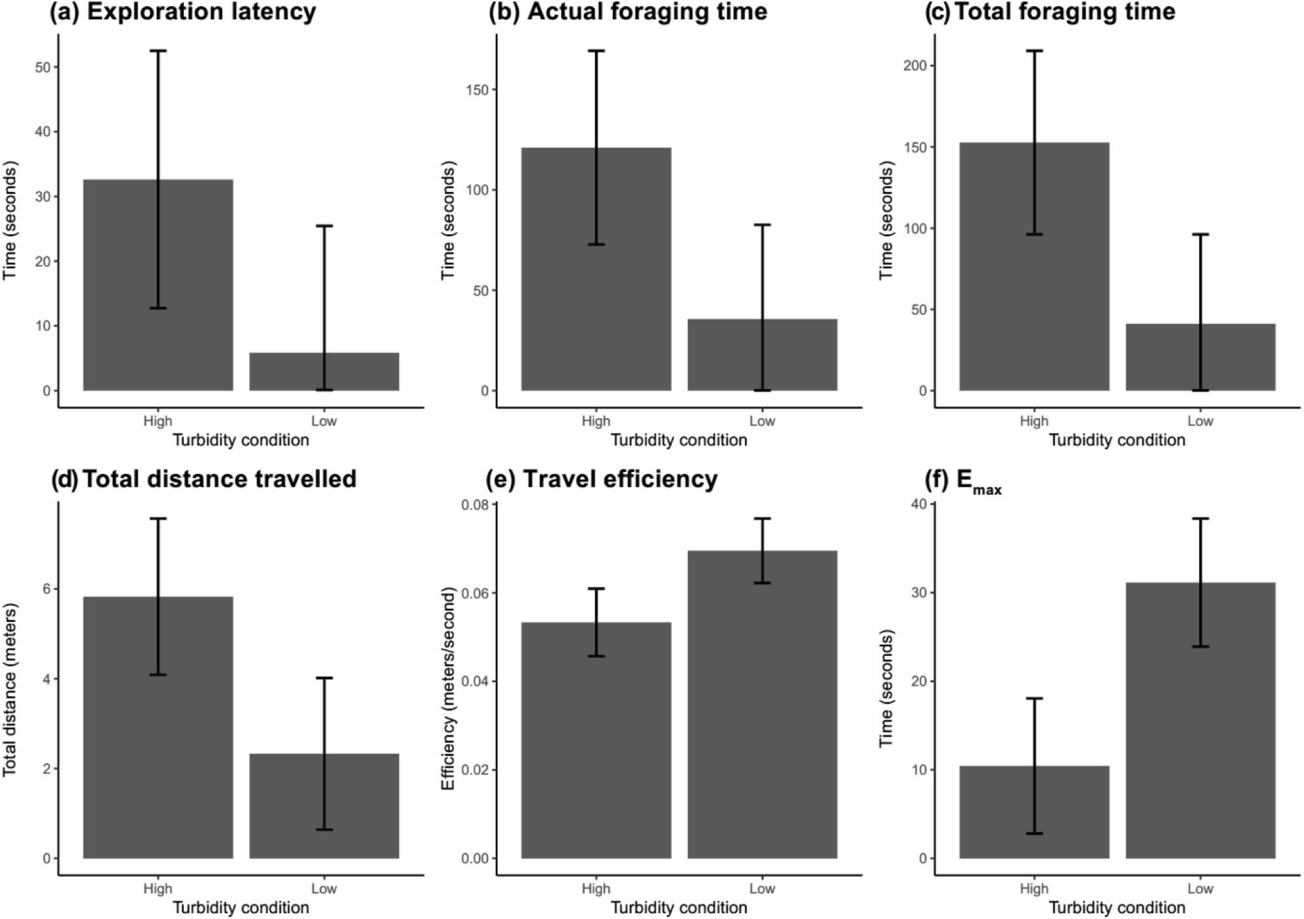
The panel shows the mean results across the six test parameters used to describe movement behaviour during foraging. Bars indicate 95% confidence intervals calculated from a linear mixed effects model. To make the visual representation of the data more intuitive, the y-axis estimates for the variable of interest is shown for the mean effect of trial rather than the intercept. (**a**) Exploration latency: the time from when the door to the refuge area is opened, and when the fish enters the test area. (**b**) Actual foraging time: the time from when the fish enters the test area to when it has eaten all six pellets. (**c**) Total foraging time: the time from when the door is opened to when the fish has eaten all six pellets (exploration latency + actual foraging time). (**d**) Total distance travelled: the distance travelled from when the fish enters the test area to when all pellets are consumed. (**e**) Travel efficiency: the total distance travelled divided by the actual foraging time. (**f**) E_max_: The maximum expected displacement where greater values indicate a straighter trajectory.

The results of the four large fish indicate that turbidity can have a significant impact on foraging and activity levels (Fig. 1). In all response variables measured, models with the turbidity treatment predicted our response variables significantly better than null models. Estimates from the full model were thus retained. For these, the time taken and distance travelled were all greater in the high turbidity treatment: exploration latency (p = 0.002) was 26.78 (95% CI: 10.49–43.07) seconds longer; total foraging time (p < 0.0001) was 111.52 (95% CI: 80.49–142.55) seconds longer; actual foraging time (p < 0.0001) was 85.40 (95% CI: 58.78–112.02) seconds longer; total distance travelled (p < 0.0001) was 3.50 m (95% CI: 2.49–4.51) greater in the high turbidity treatment. Travel efficiency (p < 0.0001) decreased by 0.016 (95% CI: 0.01—0.02) meters/second; and finally E_max_ (p < 0.0001) was 20.70 (95% CI: 14.61—26.79). Thus, in the high turbidity treatment, fish were slower, gained food less efficiently and travelled further, with a more tortuous trajectory.

### Movement analysis

**Figure 2:**
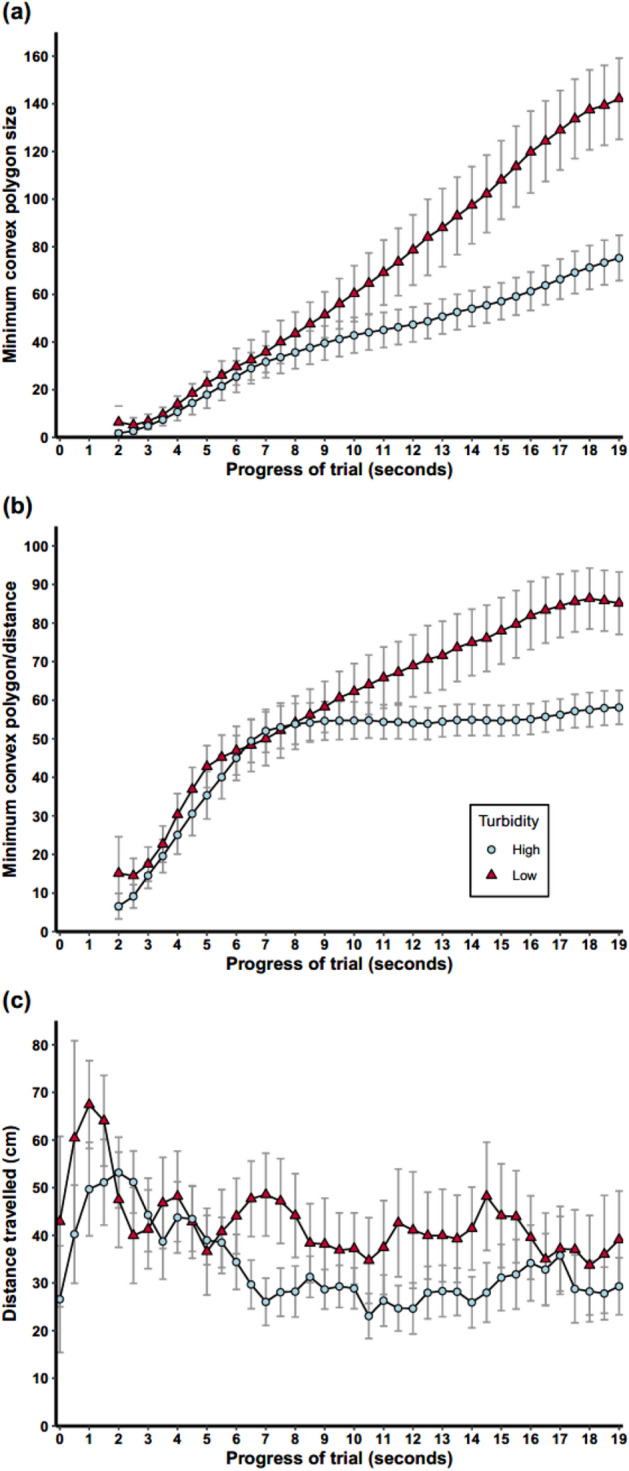
Plots show the increase in the minimum convex polygon made by the fishes’ cumulative movement up to each time point in high turbidity (blue circles) and low turbidity (red triangles) treatments, both as a raw area in m^2^ **(a)**, and as the area divided by the distance that the fish had travelled to that point in m^2^ per cm travelled **(b)**, to give a measure of how fish were converting movement into new areas explored in the tank. **(c)** The mean distance travelled (cm) measured at half second intervals, throughout the first 19 s of the experiment. Error bars indicate 95% confidence intervals.

Our analysis clearly shows that trajectories are longer and more tortuous in high turbidity conditions. A more detailed analysis of the trajectories (See Supp. Fig. 3) using the minimum convex polygon measure, indicated that there was also a significant difference in how space was explored between the two treatments. For the first 19 s of the trial, the minimum convex polygon increased significantly faster in the low turbidity treatment (Fig. 2a: Likelihood ratio test between full model and model with treatment*time interaction dropped: *X*^2^ = 295.28, p < 0.00001), and did so even when accounting for distance travelled (Fig. 2b). At the beginning of the experiment, new areas were explored quickly, which is indicated by a high cumulative rate of exploration. As the experiment progresses, there were fewer new areas to explore, and hence rates of exploration reached an asymptote, and fall when divided by time. From the MCP analysis, we can see that the fish in high turbidity spent time exploring areas either that they had already been to, or been close to, than the low turbidity group. These results indicate that fish in the high turbidity treatment spent more energy searching areas that they had visited already (Likelihood ratio test between full model and model with treatment*time interaction dropped: *X*^2^ = 131.76, p < 0.00001). They also moved 3.2 cm less per half second interval throughout the trial than fish in clear water (Fig. 2c: Likelihood ratio test between full model and model with treatment dropped: *X*^2^ 40.79 = , p < 0.00001).

**Figure 3:**
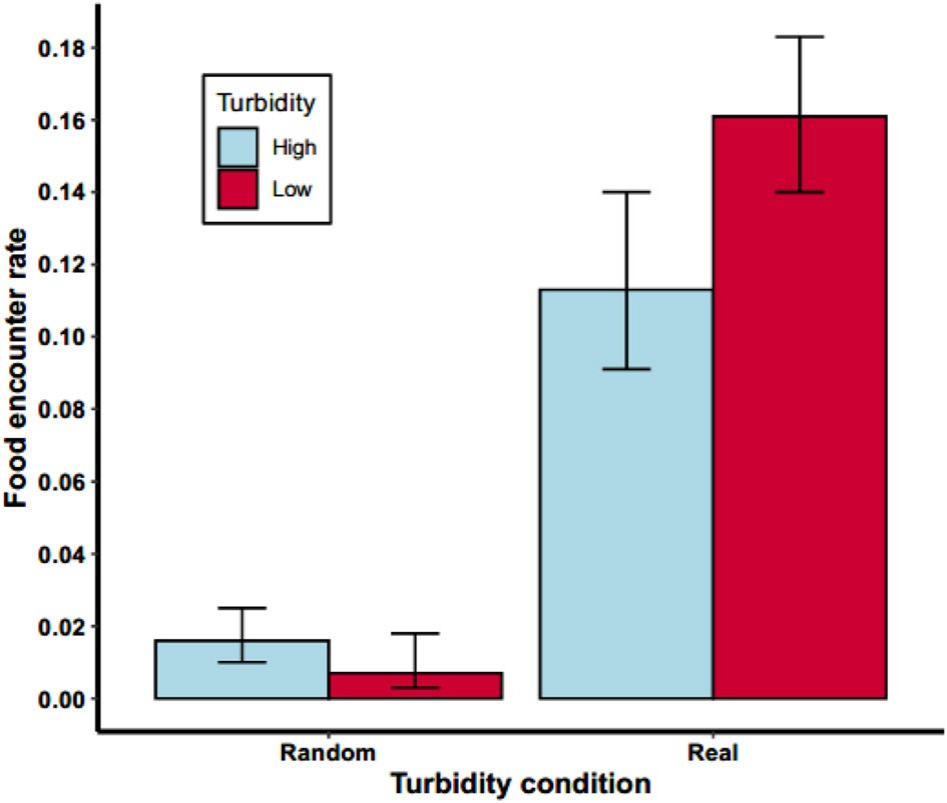
Expected food encounter frequency of simulated fish and real food encounter rate. Simulated fish were created by pseudo-randomizing the turning direction and starting direction of real fish trajectories. 95% confidence intervals, calculated from the standard error of estimates from our binomial mixed effects model are shown.

Comparison of the movement trajectories of fish to the simulated fish showed that in both treatments, fish encounter food far more often than is predicted by chance. Food encounter rate in simulated fish, estimated from our binomial GLMMs (accounting for repeated measures from individuals), was 0.016 (95% CI: 0.010—0.025) and 0.007 (95% CI: 0.003—0.018) encounters per time step in the high and low turbidity treatments respectively, whereas for real fish trajectories it was 0.113 (95% CI: 0.091—0.140) and 0.161 (95% CI: 0.140—0.183) encounters per time step (Fig. 3). Hence, even in high turbidity, fish movement was sufficiently organised to find food much better than by random searching.

## Discussion

Our results indicate that turbidity has a considerable impact on the movement of a coral reef fish species while foraging, in terms of distance travelled, time taken to find all food, and the efficiency with which fish cover space to access a resource. It not only took fish in high turbidity longer to find food, but they also spent more energy searching as routes were longer and less direct, and fish were more likely to search the same area again. We also observed that it took longer for fish to leave the shelter area and enter the more open test area. For some species of juvenile fish increased turbidity can be beneficial as it likely reduces their predation risk, allowing the fish to use shelter less ^35,72^ and increase time spent in open water ^37^. However, the fact that fish are away from their shelter longer may also lead to indirect costs such as increased predation risk and loss of territory ^64,73^. Our results suggest that turbidity has a negative impact on adult *R. aculeatus* as they are likely to be slower to leave their shelter and require more energy to locate the same number of food items. This may be particularly problematic given that an increase in energy consumption during foraging can directly lead to decreased growth rates in some species of fish^54^. We also found that the differences in mean foraging time (both total and actual) and distance travelled across treatments are quite large (total foraging time: 1.8 min; actual foraging time: 1.4 min; total distance travelled: 3.48 m) given the relatively small scale of the testing aquarium. Under real conditions, the distances over which this species is travelling while foraging is much greater (~ 10 m^2^, CN personal observation at Lizard Island, Australia). As a result, it seems reasonable to expect the consequences of turbidity on wild populations to be considerably greater.

As previously discussed, the foraging efficiency of fish in high turbidity decreased as they travelled farther and took longer to complete the task. To understand more about the underlying mechanisms of these differences, we analysed the characteristics of the trajectories. Changes in efficiency could have been achieved two ways, either fish could swim faster, or they could swim more directly to targets. We found that not only was the route longer for fish in high turbidity, but the E_max_ was higher for fish in high turbidity indicating that their paths were generally straighter. While this would typically indicate a more efficient route, in our foraging task, food sources were lined up close to each other and collecting all of the food required travelling short distances and frequent turning. Therefore, the straighter paths of fish in high turbidity reflects that they were spending more time searching areas without finding food. In addition, the fish in high turbidity were more likely to search the same area more than once, which again decreases efficiency. Rather than increasing their speed to make up for this searching redundancy, the fish in high turbidity actually swam slower throughout the trial.

The changes in behaviour that we observed are particularly compelling because non-visual cues could have been used to guide foraging behaviour. For example, extra-visual spatial cues combined with visual or mechanical cues (e.g. tank walls) from within and outside the tank could be used for orientation to at least the general area of the food rewards. As long as fish could generally orient to the correct area, once one pellet was found, one might expect finding all six pellets to be relatively straightforward. Other sensory information could also have acted as orientation cues as both olfaction^74^ and audition^17^ are involved in fish navigation, particularly over long distances. Zebrafish have been shown to change their preference for visual stimuli to olfactory stimuli after six weeks in turbid water^45^, suggesting that this is possible for fish, although the timescale of exposure between this experiment and that with zebrafish are not comparable. As the food pellets were in the water, olfactory cues were available and could be used to navigate directly to the pellets, depending on the olfactory sensitivity of the fish. Auditory and flow (from pumps) cues could also have provided less direct orientation cues. It is highly likely that the fish did use some of these cues, or others that we have not thought of, to locate the food. Comparisons of our real trajectories to simulated ones based on random movement, showed that in both treatments, fish encountered food at a much higher rate than simulated fish would. Therefore, even in high turbidity, the fish were still directing movement towards finding food, they were simply much less efficient. This suggests that while the detection distance of targets was decreased, either vision (at a reduced distance), or some other sensory system was still able to provide some location information, providing considerable improvement from random search. However, it is clear that alternate sensory systems do not provide a complete solution to the problem of sensory pollution in the visual system and it should not be assumed that they can fully compensate for the loss of visual important information.

The Picasso triggerfish may be particularly reliant on visual information, and it is possible that visual pollution would have less of an effect on other species. However, on clear shallow coral reefs where transmission of visual signals is generally reliable^22^, many species use vision to guide a wide range of behaviours (e.g. individual recognition^75^, signalling^76^, spatial learning^61^). It therefore seems reasonable to assume that other species will be affected by turbidity, even if the effect size is variable. This will be particularly true for behaviours in which vision has primacy. To unravel the impacts of turbidity on fish, we need to understand the role of multimodal sensory perception in dealing with shifts in the visual environment. However, our results indicate that it is unlikely that fish can simply use another sensory system when visual signals become unreliable and maintain the same levels of efficiency.

Initially eight fish were used for this experiment, however four small fish were excluded as they never acclimated to the experimental setup. It is tempting to ignore the behaviour of the small fish given that we have no data to analyse, however, it is worth noting this effect. We speculate that body size could influence individual behaviour such as boldness and willingness to explore, in this species. There is some evidence that body size can impact these characteristics in fish^77^, although results showed that larger fish were slower to emerge from cover in the particular species (*Brachyraphis episcopi*) and context tested. If body size does have a consistent effect on movement in *R. aculeatus*, there may be multiple strategies within the same species for dealing with changes to the available sensory information; but this is just conjecture and requires further experimentation. The emergence of two groups within our experiment, fish that participated and those that didn’t, calls into question the generalisability of our results at the population level. However, this suggests that a response to turbidity is that fish may not leave shelter to forage. This is consistent with our overall conclusion that increases in turbidity can lead to a decrease in foraging efficiency.

The levels of turbidity used in our experiment were based on what has been measured under normal conditions within the habitat range of our model species. The fact that these levels were enough to produce a stark behavioural change provides more evidence that high, but realistic, levels of turbidity might constitute a significant pollutant for fish, affecting many aspects of their life history. While fluctuations in turbidity are natural, animals may not be adapted to the more extreme and persistent turbidity caused by human activities. Given the number of threats already experienced by reef inhabitants, including ocean warming, acidification, coral bleaching and loss of habitat, it is imperative that the frequency of other possible stressors is reduced^78^. It is therefore both important and urgent to further explore the role turbidity, and particularly human-induced turbidity, on the behaviour of coral reef animals. Understanding the extent of the impact of turbidity on wild populations, whether animals possess mechanisms to cope at least for certain behaviours, and how more extreme turbidity will impact animals, will improve our understanding of how changes to sensory system input impacts behaviour. It will also provide much needed data to help scientists model and assess the impacts of turbidity on coral reef ecosystems^20^. Future studies on the effects of turbidity and the levels that can be tolerated could be crucial to conservation efforts.

## Supplementary Information

Supplementary Information 1.

Supplementary Information 2.

Supplementary Information 3.

Supplementary Video 1.

Supplementary Information 4.
