# Supplementary figures and images for: High turbidity levels alter coral reef fish movement in a foraging task
Source: Sci Rep. 2021 Mar 19;11:5976. doi: 10.1038/s41598-021-84814-5 (PMC7979735; doi:10.1038/s41598-021-84814-5)

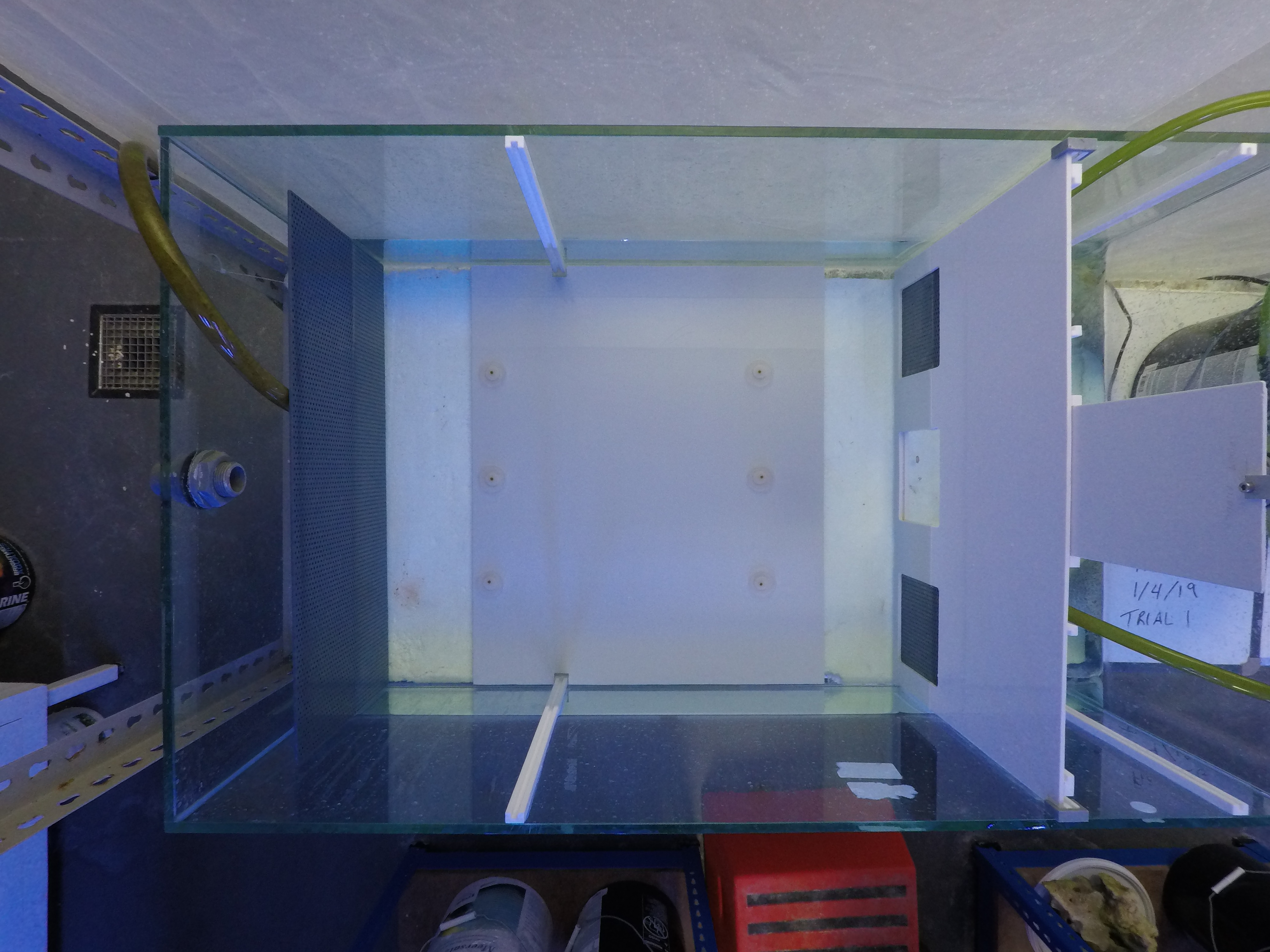

Supplement: Supplementary file 1 — Supplementary Information 1. [file 41598_2021_84814_MOESM1_ESM.jpg]

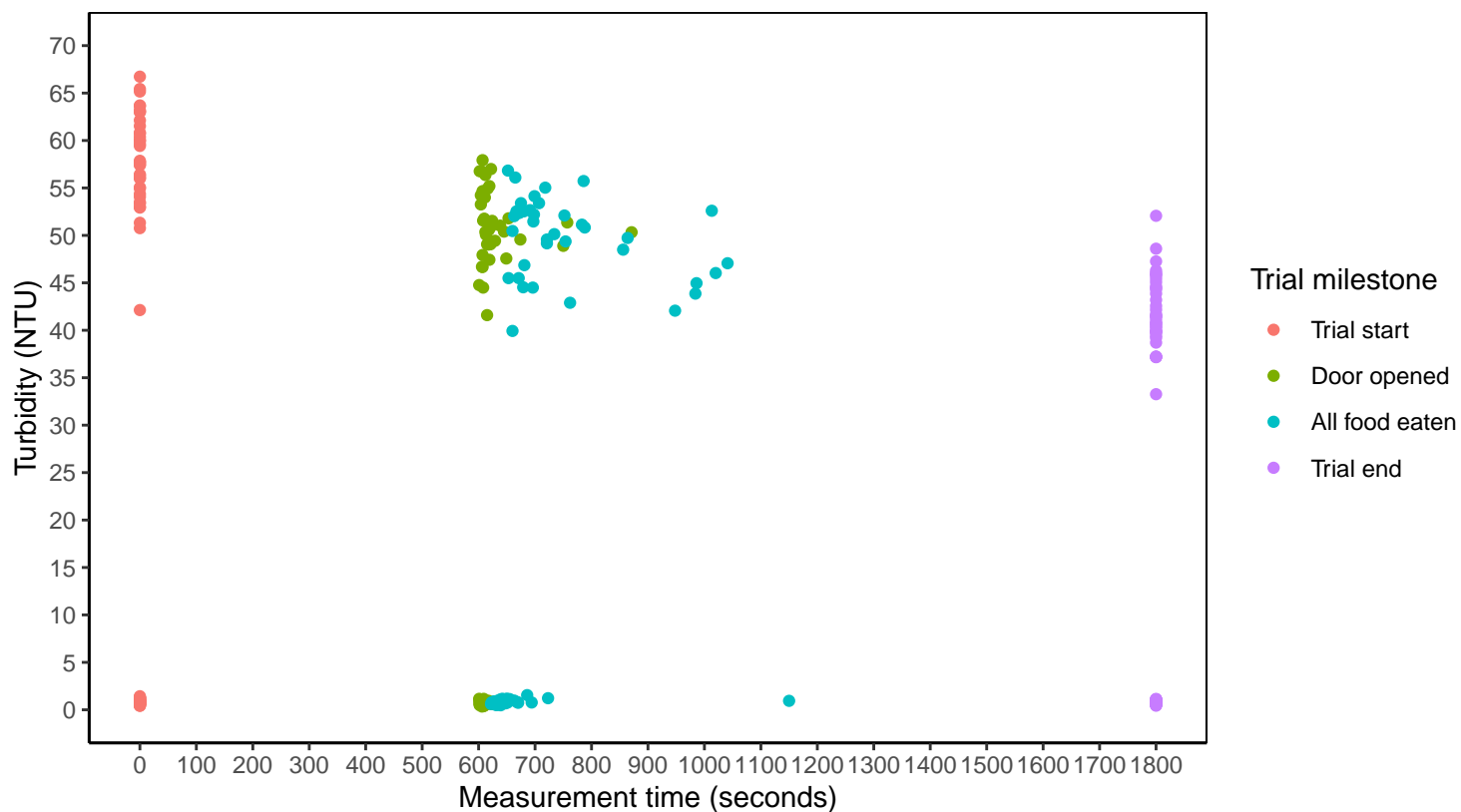

Supplement: Supplementary file 2 — Supplementary Information 2. [file 41598_2021_84814_MOESM2_ESM.pdf]

**LOW**

**HIGH**

FISH 38I

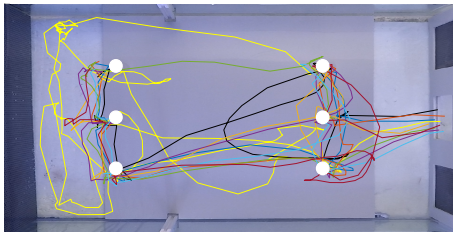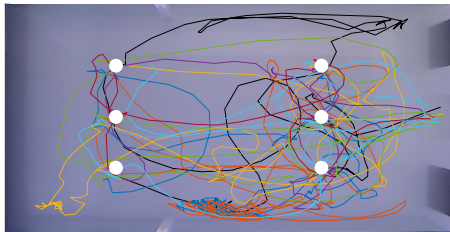

FISH 39J

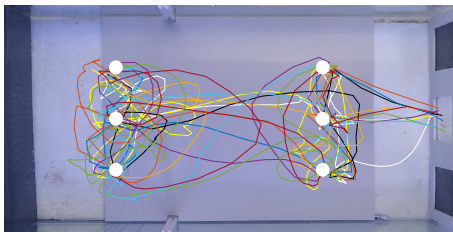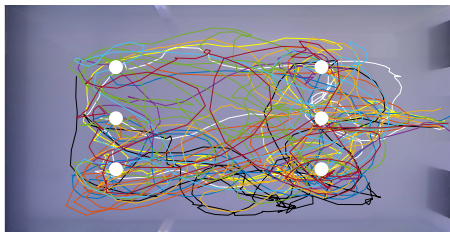

FISH 40M

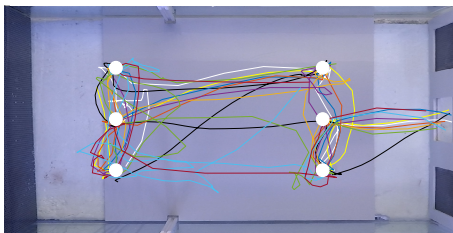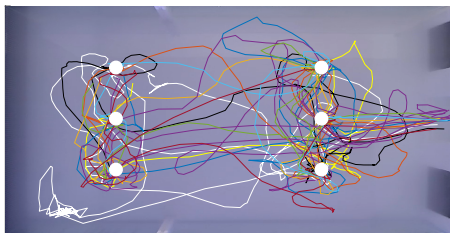

FISH 41N

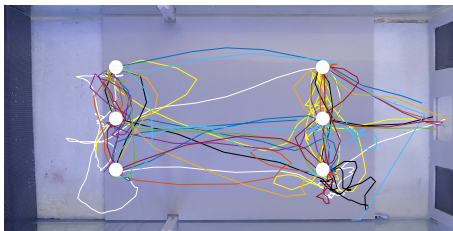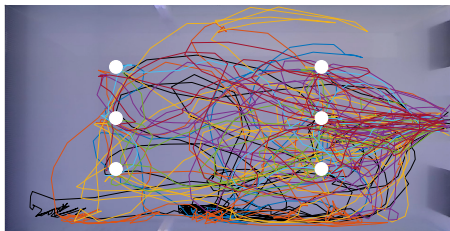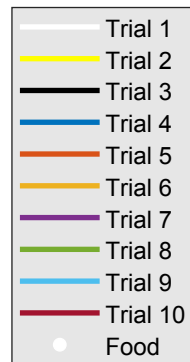

Supplement: Supplementary file 3 — Supplementary Information 3. [file 41598_2021_84814_MOESM3_ESM.pdf]
