# Supplementary material for: High turbidity levels alter coral reef fish movement in a foraging task
Source: Sci Rep. 2021 Mar 19;11:5976. doi: 10.1038/s41598-021-84814-5 (PMC7979735; doi:10.1038/s41598-021-84814-5)
Supplement: Supplementary file 5 — Supplementary Information 4. [file 41598_2021_84814_MOESM5_ESM.docx]

**SUPPLEMENTAL FIGURES**

***Supplemental Figure 1***

Title: Photograph of the experimental arena

Caption: A photograph of the experimental arena shows the main tank area and the guillotine door leading to the refuge area. Recirculating water flow is created by the two smaller green hoses on the right which bring water into the tank and the larger green hose on the left that removes water. The dark grey panel areas are perforated to allow water to flow through these areas more easily.

***Supplemental Figure 2***

Title: Experimental turbidity levels

Caption: Mean turbidity in Nephelometric Turbidity Units (NTU) measured throughout each trial for all fish.

***Supplemental Figure 3***

Title: Illustration of swimming trajectories

Caption: Swimming trajectories of fish for each trial in low (left column) and high (right column) turbidity treatments. White circles indicate the location of food pots. Trajectories were only recorded for the test area and not in the refuge

***Supplemental Video 1***

Title: Video showing the relationship between the minimum convex polygon and trajectory

Caption: (a) The value of the minimum convex polygon (MCP) by size is graphed to reflect the building trajectory. (b) A video of the progress of a trial and the building of the analysed trajectory.
